# Preliminary pharmaceutical development of antimalarial–antibiotic cotherapy as a pre-referral paediatric treatment of fever in malaria endemic areas

**DOI:** 10.1016/j.ijpharm.2014.04.023

**Published:** 2014-07-01

**Authors:** Alexandra Gaubert, Tina Kauss, Mathieu Marchivie, Boubakar B. Ba, Martine Lembege, Fawaz Fawaz, Jean-Michel Boiron, Xavier Lafarge, Niklas Lindegardh, Jean-Louis Fabre, Nicholas J. White, Piero L. Olliaro, Pascal Millet, Karen Gaudin

**Affiliations:** a Université de Bordeaux, EA 4575 Analytical and Pharmaceutical Developments Applied to Neglected Diseases and Counterfeits, Bordeaux, France; b Université de Bordeaux, FRE 3396 CNRS Pharmacochimie, Bordeaux, France; c Université de Bordeaux, Laboratory of Organic and Therapeutic Chemistry, Pharmacochimie, Bordeaux, France; d EFS (Etablissement Français du Sang) Aquitaine Limousin, Bordeaux, France; e Faculty of Tropical Medicine, Mahidol University, Bangkok, Thailand; f OTECI (Office Technique d’Etude et de Coopération Internationale), Paris, France; g Centre for Tropical Medicine, Nuffield Department of Medicine, University of Oxford, UK; h UNICEF/UNDP/WB/WHO Special Program for Research & Training in Tropical Diseases (TDR), Geneva, Switzerland

**Keywords:** Artemether, Azithromycin, Malaria, Acute respiratory infections, Pediatric, Rectal route

## Abstract

Artemether (AM) plus azithromycin (AZ) rectal co-formulations were studied to provide pre-referral treatment for children with severe febrile illnesses in malaria-endemic areas. The target profile required that such product should be cheap, easy to administer by non-medically qualified persons, rapidly effective against both malaria and bacterial infections. Analytical and pharmacotechnical development, followed by *in vitro* and *in vivo* evaluation, were conducted for various AMAZ coformulations. Of the formulations tested, stability was highest for dry solid forms and bioavailability for hard gelatin capsules; AM release from AMAZ rectodispersible tablet was suboptimal due to a modification of its micro-crystalline structure.

## Graphical abstract

**Figure:**
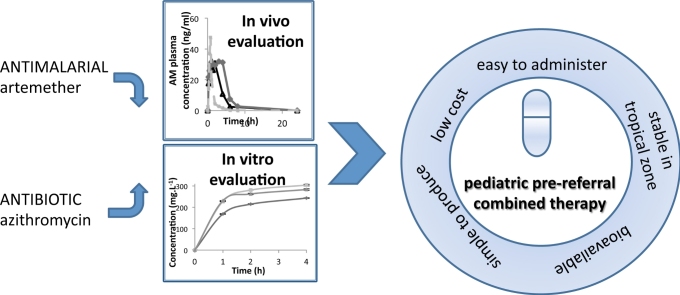

## 1 Introduction

According to recent WHO (world health organization) data (WHO, 2012a), 6.9 million of children under the age of five died in 2011. The risk of dying for a child is highest in the neonatal period, and about 16.5 times higher for children under five in sub-Saharan Africa compared to children in developed regions. WHO estimates that more than half of these early child deaths are caused by conditions that could be prevented or treated with existing simple, affordable interventions.

Pneumonia and malaria together cause the majority of deaths from infectious diseases, representing respectively 17% and 7% of deaths of children under five. Malaria deaths are caused by *Plasmodium falciparum*, and two-thirds of the pneumonia deaths are caused by *Streptococcus pneumoniae*; as a result these two organisms kill almost two million children each year in tropical countries (WHO, 2012a).

The difficulty in distinguishing severe malaria with acidotic breathing from pneumonia in children has been well documented (Bergman et al., 2004; Hoban et al., 2001; O’Dempsey et al., 1993, 1994). In general malaria is overdiagnosed and severe bacterial infections are underdiagnosed. Clinical and pathology studies point to the diagnostic uncertainty and considerable overlap between severe malaria and sepsis. In up to 20% of children dying with an in-hospital diagnosis of cerebral malaria, a different cause is found at autopsy (Taylor et al., 2004) and both conditions coexist in 15–20% of cases (Berkley et al., 2005).

Therefore correct diagnosis and treatment cannot be expected in villages and rural health centres where most cases occur. The integrated management of childhood illnesses guidelines, created in 1992 by UNICEF and WHO, recommend presumptive malaria treatment for all children with fever higher than 37.5 °C in malaria-endemic areas and that both antibiotics and antimalarial drugs are given to seriously ill febrile children as pre-referral treatment of malaria or bacterial sepsis.

To prevent death, treatment must be available near home, outside hospitals, and as close to the village or household as possible. Malaria and other febrile illnesses are frequently treated at home in malaria endemic areas (Dolecek et al., 2008; Greenwood et al., 2007). When oral treatment is no longer possible, and where injectable medications cannot be safely administered, rectal formulations can be administered by unqualified persons like parents or health volunteers. While rectal artesunate has been shown to be an effective pre-referral treatment for malaria (Gomes et al., 2009), were both malaria and pneumonia are prevalent, a combined antibiotic–antimalarial treatment is necessary. Treating malaria only might delay specific treatments of other febrile diseases with similar symptoms, such as sepsis and pneumonia (Whitty et al., 1999). Currently, systemic large spectrum antibiotics are available as oral or injectable formulations – of which none is suitable for pre-referral treatment in pneumonia and malaria endemic regions. A fixed-dose antimalarial–antibiotic coformulation would be highly desirable and practical and was thus investigated.

The minimal characteristics of the target product profile (TPP) were: (i) active principles: well-known, in-use; physico-chemical compatibility between active ingredients when co-formulated; (ii) formulation: uncomplicated, easy to scale-up, inexpensive to manufacture; well-known, inexpensive excipients; rapidly bioavailable; (iii) product development: simple, rapid, inexpensive; (iv) stability: suited to tropical conditions; (v) price: low-cost (both cost-of-goods and final product); (vi) efficacy: rapidly acting antimalarial and broad-spectrum antibiotic (to include main bacterial species causing sepsis and pneumonia in neonates, infants, toddlers and children), with as little resistance existing as possible; (vi) safety: proven general safety profile of active ingredients. The preliminary, pre-clinical development of such formulation was described in this article.

## 2 Material and methods

### 2.1 Material

Artesunate (AS), AM, AZ and dihydroartemisinin (DHA) were purchased from Knoll BASF Pharma (Liestal, Switzerland), Sanofi (France), Pfizer (USA) and Sigma–Aldrich (Saint-Quentin Fallavier, France), respectively. Excipients were of pharmaceutical grade. Sodium laurylsulfate (SLS), colloïdal silica (Aerosil 300), sodium croscarmellose, polyvinylpyrrolidone (PVP), talc and magnesium stearate were pourchased from Cooper (France). Different grades of PEG (polyethylenglycol) were purchased form Fagron (France). Microcrystaline cellulose (Avicel PH302) was purchased from FMC Biopolymer (Ireland). Betain and Lutrol were purchased form VWR (france) and BASF (Germany) respectively. Analytical solvents, acetonitrile and methanol were isocratic HPLC grade, purchased from Prolabo VWR (Leuden, Belgium). KH_2_PO_4_ and Na_2_HPO_4_, 12H_2_O were from Merck (Darmstadt, Germany).

### 2.2 HPLC analysis and method optimization

#### 2.2.1 HPLC conditions

The liquid chromatography system consisted of a Spectra System P4000 pump, a UV 6000LP detector with a cell path length of 50 mm, an AS 3000 autosampler and a SN 4000 system controller from TSP (Courtaboeuf, France). Column was Luna C8 (2) 100 A EC 5 μm, 150 × 4.6 mm (Phenomenex, France) thermostated with 560-CIL (Cluzeau Info Labo, Saint Foy la Grande, France). The flow rate was 1 mL min^−1^. The sample injection volume was 5 μL.

For method optimization, several mobile phases and detection wavelenghts were screened (Tables 1 and 2).

#### 2.2.2 Solubility study of AM and AZ in presence of lutrol

In a dissolution tester three bowls were filled with 1 L of phosphate buffer 15 mM pH 8 to simulate rectal pH conditions. Accurately weighed amounts of approximately 400 and 300 mg of AZ and AM were respectively added to each vessel. Lutrol was added to two of the three vessels at 2% and 5% of tablet mass (1.4 g). Samples were taken at time zero and at time points of 1 h, 2 h and 4 h. Homogeneity of solutions was tested by sampling each time point twice.

Determination of active pharmaceutical ingredient (API) content was obtained by calibration curves of AM and AZ at five different concentration levels (10%, 40%, 70%, 100% and 120%) in presence or absence of lutrol at 5%. Stock solution for each concentration level of AZ and AM were prepared by dissolving an accurately weighed amount of each drug and/or excipient in 50 mL of mobile phase then filtrated in a 0.45 μm filter. One milliliter of the filtrated solution of each concentration level was diluted with phosphate buffer pH 8 up to 20 mL. A 100% standard solution contained 400 and 300 mg L^−1^ of AZ and AM, respectively. These values were based on the theoretical tablet drug content. All calibration curves were proven to be linear. Furthermore, no difference was observed in presence or in absence of lutrol at 5%.

### 2.3 Formulations tested

#### 2.3.1 Compatibility study between AZ and AM

Compatibility study between both APIs, namely AZ and AM, was performed. Binary physical mixture of precisely weighted APIs was mixed using Turbula (France) at 67 rpm for 10 min. The mixture was divided into three parts, T0, ambient condition and accelerated (40 °C) condition. Samples were taken up till 3 months and analyzed using HPLC method as described.

AM and AZ compatibility was further evaluated using differential scanning calorimetry (DSC). DSC analysis was performed using Mettler Toledo TA controller and DSC30, (Switzerland) with STAR^e^ software. DSC method consisted in a heating rate of 5 °C min^−1^ in the range of 30–180 °C. Samples of 6–8 mg were precisely weighted in aluminium pans, sealed and perforated with a pin. An empty sealed perforated aluminium pan was used as reference.

#### 2.3.2 Compatibility study between APIs and excipients

Excipients chosen were checked for their usage among the ones already used in commercial formulations with AZ and AM. Compatibility was considered acquiered for excipients commercially available with both APIs separately. In addition, Am and AZ compatibility study with lutrol and talc were carried out. Six samples of AM, AZ or both APIs were added to lutrol or talc separately, then mixed (Turbula, France) at 67 rpp for 10 min and kept at 40 °C during 3 months. Samples were taken at time zero and then at 1 month, 2 months and 3 months. AM and AZ at three different concentrations levels (80%, 100% and 120%) were prepared in presence and in absence of excipient tested. Stock solutions for each concentration level of AZ and AM were prepared by dissolving an accurately weighed amount of each drug and/or excipient in 50 mL of methanol then filtrated with 0.45 μm nylon filters. Five milliliters of the filtrated solution was diluted up to 10 mL with the HPLC mobile phase. A 100% standard solution contained 500 mg L^−1^ of AZ and AM, each. All calibration curves were linear. Calibration curves statistically not different in presence or in absence of lutrol and talc.

#### 2.3.3 Preparation of tested formulations

All tested formulations (Table 3) were prepared in our laboratory. ASAM gels were prepared either dispersing gelling excipient in the aqueous solution containing both APIs or alternatively, by preparing a blank gel and then adding successively both APIs. Gel liquefaction occurred rapidly in both cases.

PEG suppositories were prepared by dispersing both APIs in the melted PEG under stirring. Formulation was molded in suppository moulds, left at room temperature until solidification, and then leveled before taking them out of the moulds. Suppositories were stored in a desiccator.

Hard gelatin capsules (HGC) were prepared by filling the powder mixture containing both APIs, a filler and a lubricant (mixed using Turbula at 67 rpm for 10 min) into gelatin capsules (size 000), using a semiautomatic capsule filler (LGA, QB300, France).

Rectodispersible tablets were prepared by direct compression using laboratory scale alternative compressor (Korsch pressen, type EK0, Berlin, Germany).

### 2.4 *In vitro* evaluation of formulations

Common pharmacotechnical tests (uniformity of mass, tablet hardness and friability, etc) were performed following European pharmacopoeia 8.0 methods and criteria.

#### 2.4.1 Dissolution assay for AZ and AM cotherapy dosage forms

The apparatus used was paddle Sotax AT 7 (Basel, Switzerland) at 37 °C ± 0.5 °C and 50 rpm. Various media were tested (cf. result Section 3.3). Finally, a bi-phasic dissolution method, initially developed for AM tablets (Gabriëls and Plaizier-Vercammen, 2004), was adapted for AMAZ cotherapy. Briefly, 150 mL of 50 mM phosphate buffer pH 6.0 and 100 mL of isooctane were placed into teach dissolution bowel. Preliminary data showed that the pH variation between 5.5 and 7.0 did not modify AZ and AM dissolution profiles significantly. Phase separation was at the paddle level, ensuring the agitation of both phases. Each form (ballasted capsule or tablet, *n* = 6) was placed at the bowel bottom (into the aqueous phase). At defined times (0/10/20/30/45/75 min), samples of 0.5 mL buffer and 0.5 mL isooctane media were taken simultaneously at the interface using 10 μm PTFE pre-filter equipped syringes. 300 μL of organic phase was evaporated at 60 °C and re-dissolved in 1550 μL of HPLC mobile phase, then completed with 450 μL of buffer phaseand mixed before injection in HLPC system.

#### 2.4.2 Preliminary stability study

Preliminary stability study was performed on several formulations: ASAZ capsules, AMAZ tablets and capsules. Prepared batches of each formulation were divided into T0 condition, ambient condition and accelerated aging condition (40 °C, 75% RH) using in a climatic chamber (Froilabo/Frilabo, France). All forms were placed into Bakelite stoppered glass vials containing each 30 dosage forms (5 vials per condition). At defined time (0/1/2/3/6 months for capsules and 0/1/2/3 months for tablets), vials were taken for aspect, content and drug release analysis (0 and 6 months). For AM and AZ content determination, crushed tablets (*n* = 6) or capsule content (*n* = 10) was dissolved directly in HPLC mobile phase, then analyzed using HPLC method described.

### 2.5 *In vivo* bioavailability evaluation

#### 2.5.1 Animal experiments

Animal experiments were performed at Etablissement Français du Sang (EFS, Aquitaine-Limousin, Bordeaux, France; accreditation number for animal experimentation n°A33063080).

Five adult healthy New-Zealand white rabbits, provided by « Le grand Claud », (Eyvirat, Dordogne, France), were used for the pharmacokinetic study of each form. Animals were housed in individual cages in controlled temperature (18–21 °C) and humidity (40–70%) room. There were fastened for 24 h prior and during the experimentation but allowed free access to water and glucose solution (5%).

The administration of each pharmaceutical formulation was adapted individually to the rabbit body weight at 20 mg/kg of AM and AZ. AM and AZ (monotherapy) in miglyol were used as rectal control formulations.

The protocol complied with the European Community guidelines (EU directive 2010/63/EU for animal experiments) as accepted principles for the use of experimental animals and internal ethic principles.

Blood samples from peripheral ear vessels (at least 1 mL of blood per time point) were collected into heparinized plastic tubes by inserting a 22 GA I.V. catheters (BD Insyte^®^, Spain). They were collected before administration, then 12 samples in 48 h post-administration. Blood samples were kept on ice before centrifugation at 1200 g during 10 min within 30 min after collection. Only supernatants (at least 500 μL) were transferred into cryo tube and conserved at −80 °C prior analysis.

#### 2.5.2 PK sample analysis

Samples were analyzed using previously described methods for AM and AZ (Kauss et al., 2012; Tarning et al., 2012).

#### 2.5.3 PK data processing

The maximal concentration (*C*_max_), and the time to reach the maximal concentration (*T*_max_) of each formulation were taken directly from mean plasma concentration–time profile curves, whereas the area under the concentration–time curve (AUC) was calculated using trapezoidal rule. Standard deviations are given for all PK parameters. Relative bioavailability was estimated from mean AUC_0–48 h_. Data at 24 h were analyzed by comparison with previously obtained parameters (Kauss et al., 2012).

### 2.6 Powder X-ray diffraction

Crystallinity and phase identification were obtained by powder X-ray diffraction analysis (PXRD) performed on a D5005 Bruker/Siemens diffractometer using the theta/theta geometry with copper radiation. Samples were deposited on a stainless steel holder without grinding to avoid artificial compression effects. Data were collected for 2*θ* angle range of 3–40° with a step size of 0.02° at a scanning speed 0.06°/min. Mixture samples were then grinded in order to compare the grinding effect with the compression in tablets products.

### 2.7 Statistical analysis

Data were analyzed using Student bilateral paired *T* test. The difference of *p* < 0.05 was considered significant.

## 3 Results and discussion

### 3.1 TPP and the choice of APIs

Considering the abovementioned TPP, only drugs already registered and in-use were taken into account.

For malaria, artemisinin derivatives are the most potent and rapidly acting antimalarial drugs available; they are very safe and well tolerated, and there is no significant resistance yet outside areas of SE Asia (WHO, 2012b). They are the WHO-recommended first-line treatment of uncomplicated malaria as artemisinin based combination treatment, (ACTs), severe malaria and pre-referral treatment (WHO, 2010). Based on generally available information and significant experience accumulated in our laboratory on physico-chemical properties and stability of artemisinin derivatives, the choice was between AM and AS; AM was expected to have stability advantages, against the considerable experience and track record existing already on AS.

Furthermore, protecting the artemisinin derivatives against resistance when deployed is also a major concern, and ideally the companion antibiotic should also have antimalarial properties. These principles informed the choice of the antibiotic. Ceftriaxone (CFX) would be the lead candidate based on antibacterial characteristics, but has minimal pharmaceutical information (which meant a longer pharmaceutical development time), had a relatively high cost-of-goods, and importantly no antimalarial activity. Of the other two contenders initially considered, chloramphenicol was dropped for clinical (real and perceived safety issues; spectrum; resistance) and compatibility reasons (incompatible with AS in preliminary studies). Eventually, AZ was selected for further investigation as the antibiotic partner in the fixed-dose antimalarial–antibiotic combination, for its expanded antimicrobial spectrum, covering most bacteria responsible for the main causes of children mortality and including malaria’s parasite *Plasmodium falciparum*, for its improved pharmacokinetic characteristics over older macrolides, (Andersen et al., 1995; Dunn and Barradell, 1996; Langtry and Balfour, 1998; Noedl et al., 2007, 2001; Yeo and Rieckmann, 1995) and for the considerable experience gained on AZ rectal forms (Kauss et al., 2013, 2012).

### 3.2 Analytical control method development

#### 3.2.1 Combined dosage of AS and AZ

Several methods were previously developed to test various combined ASAZ formulations. Some of them were published, like gel investigation (Gaudin et al., 2008) and HGC investigation (Boyer et al., 2012) using HPLC/diode array detector (DAD), HPLC/evaporative light scattering detection (ELSD) or near infrared spectroscopy. For PEG suppository and Eudragit^®^ encapsulation investigation the HPLC/DAD was found to be specific and thus used for control quality.

#### 3.2.2 Combined dosage of AM and AZ

As part of previous work, a method had been developed for the simultaneous content determination of AZ and AM in a suppository (Gaudin et al., 2011). Beside AM and AZ, DHA, the active metabolite and main degradation product of artemisinin derivatives, needed to be dosed. Its presence was expected in low concentrations; therefore the sensitivity of its detection was studied.

Mobile phase was optimized in order to improve the column life-span. The column was a grafted silica resistant to basic pH, a Luna C8(2) which was stable in the range of pHwsfrom 1 to 10. Decreasing pHww from 8.4 to 7.0 at 25 °C led to a decrease of apparent pH of the mobile phase, AZ retention and AM–AZ resolution whereas AZ peak asymmetry was not significantly different (Table 1). In order to keep sufficient resolution between AZ and AM a value of pHww equal to 7.5 was selected. The column was then thermostated at 20 °C in order to improve method robustness. In this condition, DHA was eluted as a unique peak, improving its sensitivity, with a retention factor equal to 1.37. Table 2 shows the signal-to-noise (S/N) ratio for AM, AZ and DHA obtained at various wavelengths. S/N ratio of AM was almost constant while it decreased from 210 to 215 nm for AZ. DHA S/N ratio varied in the opposite direction, with a maximum at 215 nm. Therefore, 215 nm was selected as the optimal wavelength for the simultaneous detection of AM, AZ and DHA.

The lower limit of quantification (LOQ) for AM, AZ and DHA were 25, 25 and 50 mg L^−1^, respectively.

Method validation was then performed for rectodispersible and HGC formulations and was used for the solubility, compatibility and stability studies.

### 3.3 Preformulation of antimalarial–antibiotic cotherapy

Artemisinin derivatives, AS or AM, were tested for their compatibility with AZ in a dry powder mixture at 40 °C and were found compatible (HPLC analysis, DSC) after 6 months. All excipients were either already commercialized in AZ and AM or AS formulation and therefore considered as compatible, or tested for their compatibility in physical mixtures to support their choice for coformulations. As the stress degradation study of the analytical method validation, performed on APIs, had showed that AZ and AM are unstable in acidic conditions, excipients with acidic functions were discarded. The formulations retained following preliminary explorations are summarized in Table 3. Their compatibility/feasibility, preliminary stability at 40 °C/75% RH and, for selected ones, bioavailability were evaluated.

An important point of *in vitro* evaluation was *in vitro* drug release assay. AZ aqueous solubility (39 g L^−1^ at pH 7.4 and 37 °C (Pfizer, 2013)) was not limiting regarding the dose considered (421 mg AZ dihydrate). In contrast, artemisinin derivatives are characterized by their low aqueous solubility: 0.296 g L^−1^ (Kauss et al., 2010) and 0.161 g L^−1^ (Gabriëls & Plaizier-Vercammen, 2004) for AS and AM respectively at 37 °C. For dosage forms containing 300 mg of an artemisinin derivative, this solubility was insufficient to meet solubility (ideally sink, i.e. 3.0 g L^−1^, but minimally 0.90 g L^−1^) conditions of the dissolution assay. The dissolution test was developed targeting AM, since it has lower aqueous solubility than AS. Varying the buffer pH (5.5–7.5) did not result in a satisfactory increase in AM solubility. The addition of 1% w/v of SLS to the buffer at pH 7 was necessary to obtain requested AM solubility, but this changed the dissolution rate (drug release was slower for both AM and AZ) as a consequence of the increased viscosity of the medium. The Gabriëls and Plaizier-Vercammen (2004) octanol–buffer biphasic system was adopted as the best compromise between physiological conditions and pharmacopoeia specifications. Of note, as this method required that each sample was treated individually, the variance of results was inherently higher than in a conventional monophasic dissolution assay.

The solubility of each API in the other phase i.e. AM in the buffer phase and AZ in the isooctane phase was showed to be low (respectively 1.1 ± 0.2% and 1.4 ± 0.1% of considered dose). Both phases were treated separately but reconstituted in a single sample before analysis, to give the total amount of both drugs.

### 3.4 Formulation and *in vitro* characterization of antimalarial–antibiotic coformulation

Several pharmaceutical forms were considered: classical suppository form, dry forms like capsules and tablets and semi-solid forms, like rectal gels. We have already successfully developed an AZ rectal formulation (Kauss et al., 2013, 2012). The additional challenge for developing a coformulation consisted in obtaining simultaneously good stability and bioavailability of APIs (AZ and AS or AM) with different physicochemical profiles.

ASAZ and AMAZ feasibility studies (Table 3) showed lack of AS stability. ASAZ gels suffered from liquefaction as soon as both APIs were included. ASAZ PEG suppositories presented a colour change and the stability study was interrupted. AS was encapsulated in several Eudragit^®^ polymers for protection against degradation; however, the high apparent density of encapsulated AS rendered its inclusion in suppositories and HGC technically difficult. As the compatibility of a dry blend was showed, an extemporaneously reconstituted gel was considered. Such formulation required a particular device for its reconstitution/administration. A convenient, commercially available device was the one used for diazepam rectal gel (DIASTAT^®^ AcuDial™ of Valeant Pharmaceuticals North America), but the additional cost of this primary conditioning did not comply with our TPP. Accordingly, AS based coformulations were abandoned for stability (gels, suppositories, HGC) and/or cost (extemporaneously reconstituted formulations) issues in favour of AM based coformulations.

Dry AMAZ rectal formulations were selected for further optimization. First, a simple AMAZ dry powder mixture was considered to be filled in HGC. Commonly, HGC are used mainly by oral route, but could be used for rectal route as well. Two aspects were considered of particular import during optimization: the solubility of AM to be enhanced by adding surfactants, and the flowability of the powder mixture to facilitate the industrial scale up.

Several AMAZ hard capsule formulations were developed using lactose as filler, with or without SLS, or PVP. Because of HGC capacity, and in order to achieve appropriate flowability, these formulations needed to be granulated or double-granulated (necessary in case of SLS) before filling in gelatine cores. Surfactants containing formulations gave erratic, non-reproducible drug content and drug release results. These results were attributed partially to the double-granulation process and to a possible interaction between AM and SLS. Finally, surfactants were excluded from HGC formulation and microcrystaline cellulose was used as flowability-promoting filler in addition to a lubricant. Among common lubricants, colloidal silica gave the best results compared to talc and magnesium stearate (see Table 4 for final formulation).

Beside the HGC coformulation, a rectodispersible tablet was considered. To optimize the bioavailability of both APIs, the underlying rationale of this approach was to (i) avoid the shell (i.e. capsule) and consequently its disintegration delay, (ii) ensure a rapid disintegration in an environment (the rectum) with little amounts of liquids, and (iii) enhance the solubility and consequently the absorption of AM by adding a surfactant. When possible, the excipients choice was made among those used in existing commercial formulations to avoid compatibility and regulatory issues. The key points of the development were tablets’ characteristics (e.g. hardness, friability…), rapid disintegration and AM solubility enhancement. For the latter, surfactant compatibility studies were performed (cf. Table 5). PEG 6000 was excluded due to the suspected interaction of AM with PEG 1500 and 4000 in preliminary suppository formulation studies (Table 3) preventing efficient drug release. SLS gave highly variable results and Betain was associated with AM drug content decrease. Lutrol 68 was selected as surfactant for further studies of rectodispersible AMAZ tablet and no feasibility problems were noticed. Its use was validated for the optimized formulation of AMAZ rectodispersible tablets.

The characteristics of the final formulations retained for further *in vitro* evaluations are summarized in Table 5.

AMAZ tablets and capsules were evaluated for customary pharmacotechnical controls (mass and content uniformity, tablet friability, disintegration, etc.) according to the European Pharmacopoeia 8.0 requirements. As there is currently no specific monograph for rectodispersible tablets, tablet disintegration was tested following the monographs of dispersible and vaginal tablets. All the tested parameters complied with pharmacopoeia standards.

Dissolution assay and preliminary stability studies were also performed on these formulations, up to 3 or 6 months for tablets and HGC respectively (Table 6).

The preliminary stability results showed that contrary to ASAZ formulations (degradation of AS after 6 weeks), AMAZ formulations were stable for at least 3 months in accelerated conditions.

The drug release of both APIs from HGC and AZ from tablets complied with pharmacopoeia specification of conventional drug release, while drug release was low for AM from rectodispersible tablets – a surprising finding, given the addition of a superdisintegrant and a surfactant to the formulation. As the *in vitro* biphasic assay was not representative of physiological conditions regarding rectal mechanical forces, the formulation was tested *in vivo* despite *in vitro* results. Drug release of both API from HGC was not significantly different (*p *> 0.05, Student test) after 26 weeks of accelerated stability compared to the initial release.

### 3.5 *Invivo* evaluation

**Fig. 1:**
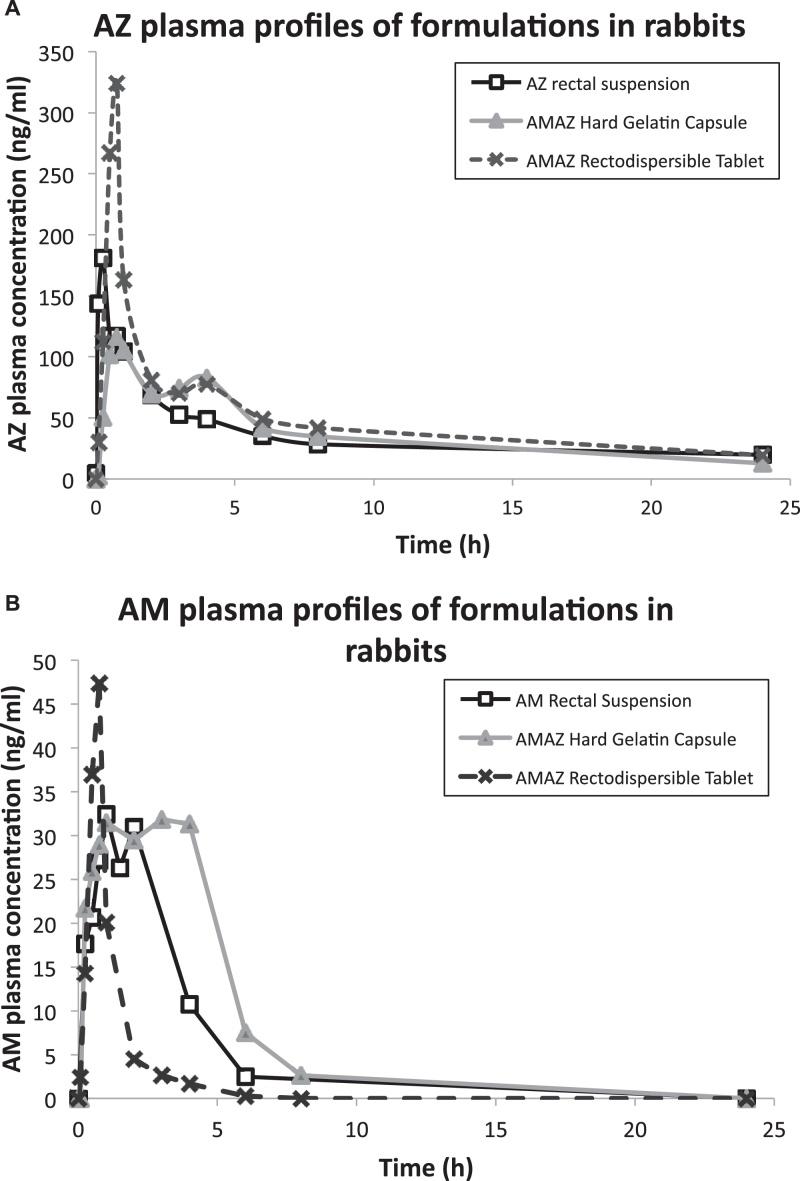
AZ (A) and AM (B) rabbit plasma profiles of various rectal formulations administered at 20 mg/kg body weight.

AMAZ coformulations were administered rectally to rabbits and their bioavailability evaluated. AZ suspension and AM solution in oily vehicles were used as rectal controls. The results are summarized in Fig. 1A and B for AZ and AM plasma profiles respectively and in Table 7 for pharmacokinetic parameters.

For ethical reasons this preliminary bioavailability study was limited to groups of five animals per experimental condition, which showed marked variability. Further animal studies containing larger groups of animals would be necessary to reduce the variability for the selected formulation, but this was beyond the scope of the current preliminary exploratory study.

AMAZ HGC formulation produced satisfactory absorption for AZ (comparable AUC and relative bioavailability F′) and delayed but enhanced absorption of AM (relative bioavailability of 146%, difference not statistically significant due to large standard deviation) compared to rectal single-agent control formulations. Compared to previously described AZ monotherapies (Kauss et al., 2013, 2012), HGC parameters were comparable (107% and 105% of relative bioavailability for AZ HGC in monotherapy and cotherapy respectively), but AZ HGC absorption was lower than the one obtained with AZ suppository (232% of relative bioavailability after 24 h) (Kauss et al., 2012). In conclusion, AZAM HGC provided AZ absorption comparable to the one obtained with rectal oily suspension, but did not enhance it. AM alone in HGC was also administered to rabbits in a monotherapy as a rectal dry form control condition and gave statistically non different results (AUC 187 ± 71 ng × h/mL, *p* > 0.05) as AM absorbed from cotherapy. Furthermore, these results demonstrated that an additional API did not diminish the absorption rate of AM or AZ form HGC.

AMAZ tablet moderately but non-significantly enhanced AZ absorption (relative bioavailability 133%, *p* > 0.05), but failed to provide sufficient AM absorption (relative bioavailability 25%, *p* ≤ 0.05). These results confirmed the ones obtained during *in vitro* evaluation. Despite the presence of a disintegrant and a surfactant, AM was not adequately absorbed in rabbits.

### 3.6 Further investigations to explain low AM release of AMAZ tablets

Unexpectedly low AM drug release from AMAZ tablets was further investigated. Possible explanations are that (i) crystalline modification of AM in tablet form diminished its drug release and its bioavailability and (ii) lutrol added as a surfactant did not significantly increase AM solubility – at least not enough to compensate the decrease induced by crystalline modification.

#### 3.6.1 X-ray characterization

Powder X-ray diffraction (PXRD) patterns were determined on powder of AZ, AM, AMAZ mixture and tablets of AZ, AM and AMAZ mixture. Each PXRD pattern presented the characteristic peaks of AZ or AM or both depending on the sample, and no spurious extra peaks.

**Fig. 2:**
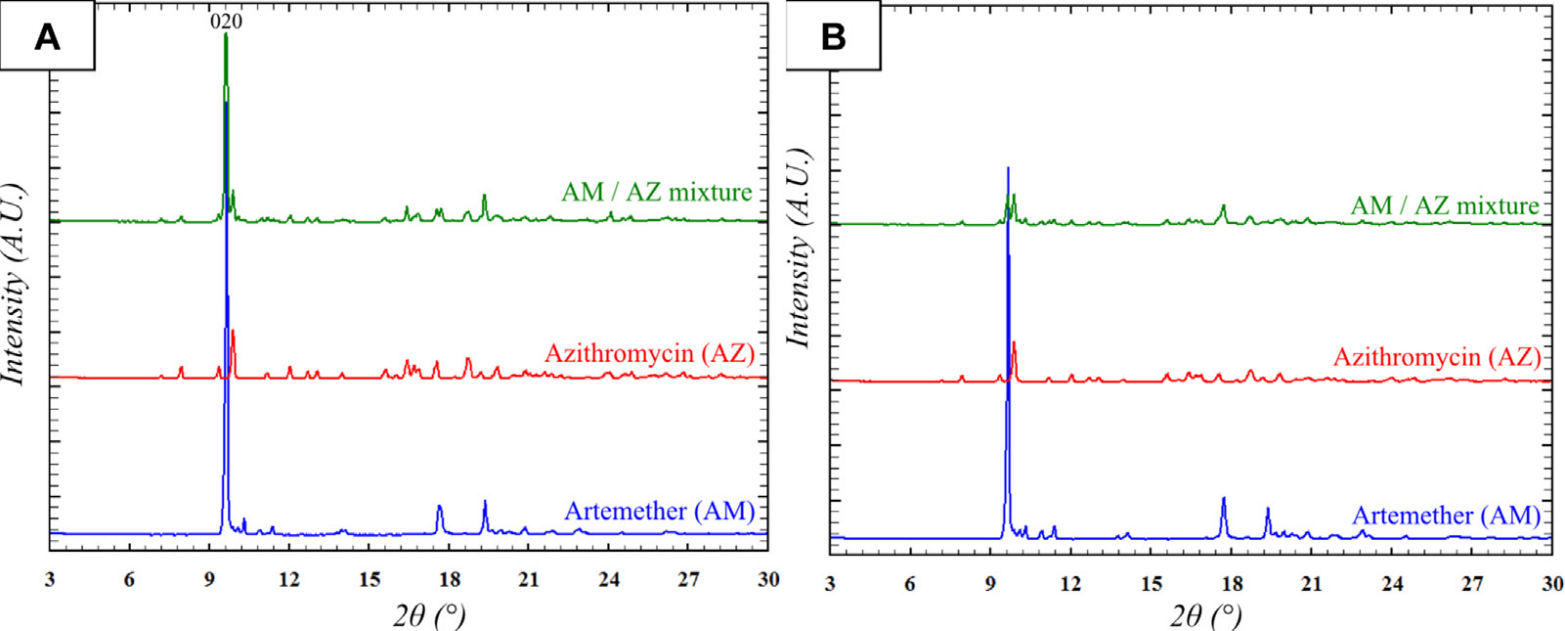
Powder X-ray diffraction patterns of AM, AZ and AM/AZ mixture of (A) powder samples and (B) compressed tablet samples.

All PXRD patterns of samples containing AM, except the AMAZ tablet, showed a strong preferred orientation effect of the AM phase as shown by the abnormally intense 020 reflection of AM (Fig. 2A), which may be due to strong anisotropy of the shape of the AM crystallites. It is worth to note that this effect, which decreased markedly in the compressed mixture of AMAZ tablets, was present in all other samples, including the compressed AM (Fig. 2B). This difference, between the PXRD pattern of the compressed mixture tablets and the others, constituted the sole distinct feature within the observed patterns.

In order to better understand this effect, a Rietveld refinement (Rietveld, 1969) was performed on each PXRD pattern. The refinement performed on the AMAZ mixture samples led to the same weight proportions of AM and AZ in the powder and the tablet samples which were estimated at 40(2)% and 60(2)% respectively – thus in good agreement with the theoretical proportions of 42% and 58%, respectively. These results allowed to exclude any chemical interaction or decomposition of AM or AZ and any amorphization during the compression.

The Rietveld refinement of pure compounds confirmed the strong effect of preferred orientations on both powder and tablet AM samples, while no significant orientation effect could be detected on AZ samples. The preferred orientations effect were of the same amplitude between AM powder and tablet samples (March-Dollase parameter ∼0.6) (Dollase, 1986), but interestingly, the Rietveld refinement on AMAZ mixture revealed a significantly different behavior: while the preferred orientations effect was still strong in the powder mixture (March-Dollase parameter ∼0.5), this effect completely disappeared in the tablet mixture (March-Dollase parameter ∼1.0). This indicated that the loss of the preferred orientations was only due to the compression of the powder mixture. According to the Rietveld results the distinct behavior of the AMAZ mixture tablets relative to the powder mixture might be related to the sole preferred orientations effect and cannot be attributed to changes in the AM and AZ proportions in the samples. The total loss of the preferred orientations effect after compression of the mixture might be due to a change of crystallites morphology, which was possibly due to either the breaking of the crystallites into smaller pieces or by the aggregation of crystallites of AM and AZ. In either case the shape of the new particles became more isotropic, thus reducing the preferred orientations. Nevertheless, the first hypothesis was contradicted by the absence of this effect in the pure AM tablet samples and by low absorption of AM tablets on pharmacokinetic measurements. Thus aggregation was a more likely explanation. This hypothesis was further supported by finding in the Rietveld refinements of a small broadening of the diffraction peaks at high theta value for the AMAZ mixture tablet sample only, which was consistent with micro-deformations of the material caused by aggregation of AM and AZ crystallites. As grinding of the AMAZ powder mixture did not lead to the suppression of preferred orientations, this aggregation would occur in tablet samples only, and might be due to local surface warming during compression.

Taken together, these results demonstrate that compression had an effect on the crystalline microstructure and could help to explain the observed differences in AM release between compressed and uncompressed forms.

#### 3.6.2 AM and AZ solubility enhancement in presence of lutrol

To better characterize the lutrol effect as a surfactant in enhancing the AM and AZ solubility, the latter was studied in the presence of 2% lutrol, at tablet dosage form quantities (300 and 400 mg for AM and equivalent anhydrous AZ, respectively).

**Fig. 3:**
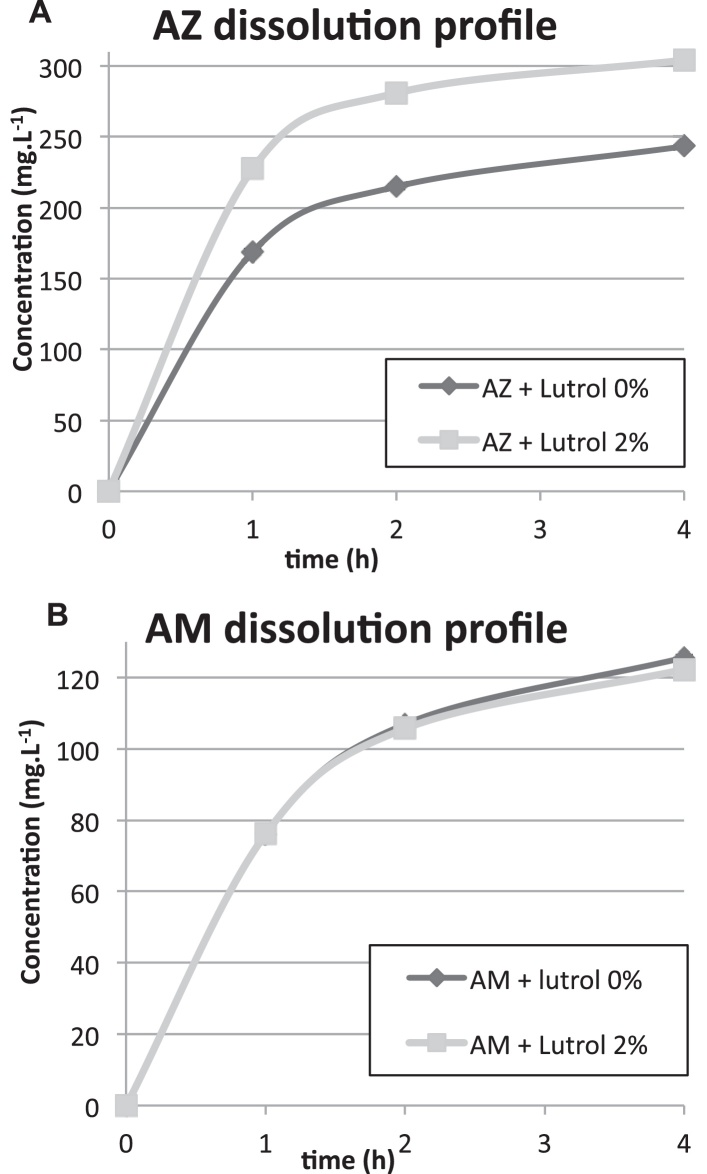
Dissolution study in the presence of lutrol for AZ (A) and AM (B).

AZ dissolution rate improved with lutrol at 2% (57% vs. 42% respectively after 1 h, *p* < 0.05). After 4 h with 2% lutrol, 76% of the expected concentration was reached while compared to 60% without lutrol (Fig. 3A). Conversely, 2% lutrol has no effect on AM solubility or dissolution rate in tested conditions (Fig. 3B). Similar results were found for the AMAZ tablet. Additional studies demonstrated that AM dissolution increased with higher concentrations (5%) of lutrol (from 25% to 30% for 0% and 5% of lutrol respectively, *p* < 0.05). Further studies are needed now on more efficient surfactants to improve AM solubility and dissolution rate on order to compensate the decreased drug release induced by compression.

## 4 Conclusions

Our study demonstrates that dry pharmaceutical forms are the most suited coformulation for an antimalarial–antibiotic combination treatment in malaria-endemic areas. They provide an easy to administer, inexpensive, stable formulation, with enhanced AM rectal bioavailability and comparable AZ bioavailability with respect to individual rectal preparations. The co-administration of individually-formulated antimalarial and azithromycin (e.g. AS rectal soft capsule Rectocap^®^ and the previously developed AZ suppository (Kauss et al., 2013)) remains a valid, shorter-term alternative.

Rectodispersible AMAZ tablets gave satisfactory AZ but low AM drug release and bioavailability, possibly due to compression-induced changes in AM crystalline micro-structure.

## Figures and Tables

**Table 1:** Comparison of pH mobile phases composed by 80% of CH_3_OH and 20% of phosphate buffer (30 mM).

pwwHof buffer	pwsHmobile phase^a^	*k*_AZ_	*k*_AM_	Asymmetry of AZ peak	Rs_AM/AZ_
8.4	10.3	4.48	3.15	1.13	2.27
8.0	10.2	4.34	3.12	1.11	2.76
7.5	10.0	4.21	3.14	1.12	2.49
7.0	9.4	4.01	3.19	1.10	1.67

a Measurement performed at room temperature (i.e. 22 °C, air conditioned).

**Table 2:** S/N ratio of compounds at various wavelengths.

Compound	Wavelength (nm)		
	210	212	215
	S/N		
DHA 500 mg L^−1^	72	92	100
AM 350 mg L^−1^	28	31	30
AZ 500 mg L^−1^	220	201	172

**Table 3:** Summary of tested formulations for rectal administration of antimalarial–antibiotic cotherapy and conclusions.

APIs	Formulation		Immediate compatibility	Stability	Animal pharmaco-kinetics	State	Remarks
Artesunate + azithromycin	Gel (several formulations)		No	No	ND	No go	Gel liquefaction; could propose extemporaneous preparation type DIASTAT^®^, but high additional costs
	PEG suppo-sitory	PEG 6000 monolayer or bilayer	No	No	ND	Stopped	Eudragit^®^ encapsulated AS could be considered for PEG inclusion, however problems of formulation encapsulated drug capacity
		PEG 1500/4000	Yes	Interrupted (colour change)			
	Hard capsule		Yes	No	ND	No go	AS degradation after 6 weeks at 40°C/75% RH
Artemether + azithromycin	PEG suppository		No	ND	ND^b^	Stopped^b^	Problems of drug release consecutive to AM-PEG interaction (shell formation); 0.1% of LS^a^ in dissolution medium or 1% LS^a^ in suppository are necessary for *in vitro* drug release.
	Hard capsule	With LS^a^	No	No	ND	No go	LS^a^–AM interaction Double granulation necessary
		Without LS^a^	Yes	Yes	Yes	Potential candidate	
	Rectodispersible tablet		Yes	Yes	Low AM absorption	No go	Low absorption may be caused by aggregation between AM and AZ.

ND = not determined.

a LS = Na laurylsulfate.

b AM suppository in monotherapy gave no absorption.

**Table 4:** Final formulations for of AMAZ capsules and tablets.

API/excipient (% w/w)	AMAZ HGC	AMAZ tablet
Azithromycin 2H_2_O (anhydrous)	51.05 (48.74)	29.13 (27.79)
Artemether	36.56	20.87
Microcrystalline cellulose	12.19	41.3
Croscarmellose sodium	–	4.0
Lutrol F68	–	2.0
Colloidal silica	0.20	0.2
Talc	–	2.0
Mg stearate	–	0.5

**Table 5:** AM compatibility with surfactants after 3 months at 40 °C (HPLC analysis of AM drug content).

Mean AM content (%)		Coefficient of variation
AM + SLS	101.14	10.83
AM + lutrol F68	96.06	5.40
AM + betain	83.86	0.14
AM + PEG 6000	102.61	2.13

**Table 6:** *In vitro* evaluation of AMAZ formulations (*n* = 6, mean ± SD).

	Time (weeks)	Storage condition	AMAZ HGC		AMAZ tablet	
			AZ	AM	AZ	AM
Drug content (%)	T0	–	100.4 ± 1.3	101.2 ± 1.1	98.7 ± 1.6	97.6 ± 1.1
	12	ambient	101.7 ± 1.0	100.0 ± 0.9	101.2 ± 0.8	100.3 ± 2.7
	12	40°/75% RH	100.8 ± 0.6	99.0 ± 0.2	99.2 ± 1.8	98.3 ± 2.7
	26	40°/75% RH	97.3 ± 0.8	100.9 ± 1.5	ND	ND
Drug released at 45 min(%)	T0	–	88.0 ± 15.0	78.9 ± 11.0	22.6 ± 3.3	88.8 ± 0.8
	26	40°/75% RH	93.4 ± 3.6	87.1 ± 11.4	ND	ND

ND: not determined.

**Table 7:** AZ and AM disposition in the rabbit after administration of individual drugs as monotherapy as control (AZ or AM in miglyol) or fixed-dose coformulations (*n* = 5, mean ± SD).

	Control	AMAZ HGC	AMAZ tablet

AM parameters			
*C*_max_ (ng/mL)	35 ± 11	56 ± 18	59 ± 38
*T*_max_ (h)	1.4 ± 0.6	3.0 ± 1.2	0.8 ± 0.2
AUC _0–48 h_ (ng × h/mL)	126 ± 85	185 ± 130	46 ± 19
F′	100	146	25

AZ parameters			
*C*_max_ (ng/mL)	191 ± 49	118 ± 35	410 ± 212
*T*_max_ (h)	0.37 ± 0.32	1.25 ± 0.89	0.80 ± 0.19
AUC _0–48 h_ (ng × h/mL)	858 ± 198	898 ± 469	1141 ± 404
F′	100	105	133
